# Systematic Review on the Effectiveness of Comprehensive Behavioural Intervention (CBIT) for Tics in Children and Adolescents

**DOI:** 10.1192/bjo.2026.11193

**Published:** 2026-06-30

**Authors:** Reka Ajay Sundhar, Helen Meaden, Harm Boer, Kavita Krishnan, Suzanne Toft

**Affiliations:** 1 Black Country Health Care NHS Foundation Trust, Dudley, United Kingdom; 2 Coventry and Warwickshire Partnership Trust, Birmingham, United Kingdom; 3 Coventry and Warwickshire Partnership Trust, Leamington, United Kingdom; 4 Black Country HealthCare NHS Foundation Trust, Walsall, United Kingdom

## Abstract

**Aims:**

Tourette syndrome and chronic tic disorders are neuro developmental conditions characterised by sudden, repetitive motor movements and/or vocalisations–known as tics–that persist for at least one year. These disorders typically begin in childhood and can lead to significant distress, impair daily functioning, and reduce overall quality of life. Although pharmacological treatments and behavioural therapies are available, long-term use of medication is often associated with adverse side effects. Consequently, there is growing interest in non-pharmacological approaches. This systematic review therefore examined the effectiveness of Comprehensive Behavioural Intervention for Tics (CBIT) in children and adolescents diagnosed with tic disorders.

**Methods:**

For this review, several electronic databases were searched, including ProQuest PsycInfo, Ovid Medline, Ovid Embase, Ovid Emcare, the Cochrane Library, PubMed, NICE, and BMJ Best Practice. Searches were limited to English-language publications and to studies involving children and adolescents under 18 years of age with tic disorders who received Comprehensive Behavioural Intervention for Tics (CBIT). Eligible study designs included case reports and case series, and both quantitative and qualitative research and Mixed Methods Appraisal Tool (MMAT) was used to assess methodological quality. In total, five studies met the inclusion criteria. These studies examined the effectiveness of CBIT delivered in various formats–including group-based CBIT, tele-CBIT, and modified CBIT–in reducing tic severity among children and adolescents.

**Results:**

Two independent reviewers analysed the data and identified substantial heterogeneity, which prevented further meta-analysis. However, the findings were instead synthesised narratively under three themes: effectiveness of CBIT, wider benefits for tics and acceptability. Across studies, CBIT consistently reduced tic severity in children and adolescents. Several studies also reported broader improvements, including reduced anxiety and depressive symptoms, alongside enhanced self-esteem and cognitive reappraisal. Acceptability and patient satisfaction were high across delivery formats, with group-based, tele-CBIT, and modified CBIT demonstrating comparable outcomes to traditional face-to-face interventions.

**Conclusion:**

The review was constrained by the small number of studies and limited sample sizes, which reduced the reliability and generalisability of the findings. However, the results highlighted the effectiveness of CBIT in reducing tic severity alongside wider benefits for children with tic disorders. Thus the effectiveness, acceptability, and satisfaction across different CBIT delivery formats suggest promising potential for implementation in resource-constrained settings, with the possibility of enhancing quality of life for young people with tic disorders. Further research is needed to evaluate CBIT in combination with pharmacological treatments and neuro-modulatory approaches.

